# Inferior vena cava interruption in renal cell carcinoma with tumor thrombus: surgical strategy and perioperative results

**DOI:** 10.1186/s12893-021-01400-2

**Published:** 2021-11-21

**Authors:** Zhuo Liu, Qiming Zhang, Xun Zhao, Guodong Zhu, Shiying Tang, Peng Hong, Liyuan Ge, Shudong Zhang, Guoliang Wang, Xiaojun Tian, Hongxian Zhang, Cheng Liu, Lulin Ma

**Affiliations:** Department of Urology, Peking University Third Hospital, Beijing, China

**Keywords:** Renal cell carcinoma, Tumor thrombus, Inferior vena cava, interruption

## Abstract

**Background:**

To analyze the influence of inferior vena cava (IVC) interruption for perioperative and oncological results in patients with renal cell carcinoma and tumor thrombus and summarize the surgical strategies of IVC interruption for different situations.

**Methods:**

We retrospectively analyzed the clinical and pathological data of 103 patients in our center. Patients were divided into two groups with 32 cases (31.1%) underwent IVC interruption (Group 1) while 71 cases (68.9%) did not. For comparison of continuous variables, the Mann–Whitney *U* test was used. For comparison of categorical variables, Chi-square tests were used. A propensity score based matching method was used to eliminate possible bias. Kaplan–Meier plots were performed to evaluate the influence of IVC interruption on overall survival and cancer specific survival. All the statistical analyses were performed using SPSS 24. A P value < 0.05 was considered statistically significant.

**Results:**

Among the 32 patients who underwent IVC interruption, the median age was 61 years and the median tumor size was 7.7 cm. There were 28 males and 23 tumors were on the right side. We successfully matched 29 patients who underwent IVC interruption to 29 patients without this procedure in 1:1 ratio. No significant differences existed in baseline characteristics between the groups. The comparison of perioperative data showed that patients who underwent IVC interruption had significantly longer median postoperative hospital stays (13 vs 9 days, P = 0.022) and a higher overall postoperative complication rate (79.3 vs 51.7%, P = 0.027). According to the side and shape of tumor thrombus, it could be divided into four categories. There were 15 cases (46.9%) with right filled-type tumor thrombus (RFTT), 8 cases (25.0%) with right non-filled-type tumor thrombus (RNFTT), 1 case (3.1%) with left filled-type tumor thrombus (LFTT) and 8 cases (25.0%) with left non-filled-type tumor thrombus (LNFTT). According to different categories, different surgical procedures were adopted.

**Conclusions:**

IVC interruption will increase the incidence of overall postoperative complications, but not the risk of major postoperative complications. Tumor thrombus should be divided into four categories, and different sides and shapes of renal tumor thrombus need different operative procedure of IVC interruption.

The purpose of surgical treatment of renal cell carcinoma (RCC) with inferior vena cava (IVC) tumor thrombus is to remove tumor burden completely [1]. Invasion of IVC wall has been reported to be a risk factor of disease recurrence and poor prognosis [2]. The 5-year survival rate was only 26% in patients without resection of the invaded IVC wall, however it could reach 57% in patients with radical resection [3]. Therefore, it is necessary to resect the invaded vessel wall in patients with IVC wall invasion. Because of the differences in anatomical structure and collateral circulation between the left and right renal tumors, the surgical procedures are also different [4]. Besides, the shape, location and extent of invasion of tumor thrombus can also affect the surgical procedure [5]. To describe the clinical characteristics of patients with renal cell carcinoma (RCC) and inferior vena cava (IVC) tumor thrombus who underwent IVC interruption and to explore the effect of different sides and shapes of renal tumor thrombus on the surgical procedure of IVC interruption, we retrospectively analyzed the clinical data of 103 patients with renal cell carcinoma and IVC tumor thrombus from January 2014 to March 2019 who received IVC interruption during surgical treatment in our center.

## Object and method

### Clinical data

The clinical data of 103 renal cell carcinoma patients with IVC tumor thrombus from January 2014 to March 2019 were retrospectively analyzed in Peking University Third hospital. Patients without surgical treatment, with recurrence of tumor thrombectomy, and with pathological types of non-renal cell carcinoma were excluded. All patients underwent ultrasound of urinary system before operation to evaluate renal tumor, including lateral, location, diameter and so on. The TNM staging of renal tumors was evaluated by chest and abdominal CT scan (UICC, International Union against cancer, 2010) [6]. MRI was performed to determine the length of tumor thrombus and evaluate whether the IVC wall was invaded or not. Thrombus level was defined according to the Mayo classification system. [7]. American Society of anesthesiologists (ASA) classification was used to classify patients according to their physical status and surgical risk [8]. Patients with hemorrhagic disease, cardiopulmonary insufficiency, intolerance of anesthesia and operation were excluded.

According to the side and shape of renal tumor thrombus, it could be divided into four categories: right filled-type tumor thrombus (RFTT), right non-filled-type tumor thrombus (RNFTT), left filled-type tumor thrombus (LFTT), left non-filled-type tumor thrombus (LNFTT). The clinical features of filled-type tumor thrombus (FTT) are as follows: ①The width of the tumor thrombus was wider in contrast-enhanced CT of urinary system or enhanced MRI of inferior vena cava; ②The tumor thrombus invaded both the right and left vascular wall of IVC; ③ The distal end of the tumor thrombus was usually complicated with bland thrombus; ④ There was no blood flow between the tumor thrombus and the surrounding vascular wall in IVC vascular ultrasound. The clinical features of non-filled-type tumor thrombus (NFTT) were as follows:① The width of the tumor thrombus was narrower in contrast-enhanced CT of urinary system or enhanced MRI of inferior vena cava; ② The tumor thrombus only invaded one side of IVC (renal tumor side), but no invasion of the contralateral wall; ③ The distal end of tumor thrombus was usually not complicated with bland thrombus;④ There was blood flow between the tumor thrombus and the surrounding vascular wall in IVC vascular ultrasound.

### Operation method

IVC interruption refers to various surgical procedures that may lead to complete blockage of the IVC blood return. It mainly includes three aspects: ①The tumor thrombus invades the IVC wall extensively. The invaded vessel wall undergoes segmental resection. The distal end, proximal end of IVC and contralateral renal vein are sutured continuously with sutures or ENDO-GIA. ② The tumor thrombus invades one side of the IVC vessel wall. For example, the left renal tumor invades the left vessel wall, while the right vessel wall is not invaded. Therefore, the reflux channel from the contralateral renal vein to the proximal end of IVC is retained. The distal end of IVCis sutured. ③ Tumor thrombus may be complicated with long non-tumor bland thrombus at the distal end of the IVC. The distal end of IVC is usually sutured to avoid the potential risk of bland thrombus shedding and pulmonary embolism. In our center, we do not routinely use artificial blood vessels. First, the collateral circulation formed by compensation can ensure the blood return of the contralateral kidney, reducing the necessity of using artificial blood vessels. Secondly, artificial blood vessels may cause secondary thrombosis and increase the potential risk of pulmonary embolism. The use of anticoagulant drugs may cause a potential risk of bleeding.

#### Right filled-type tumor thrombus (RFTT)

**Fig1:**
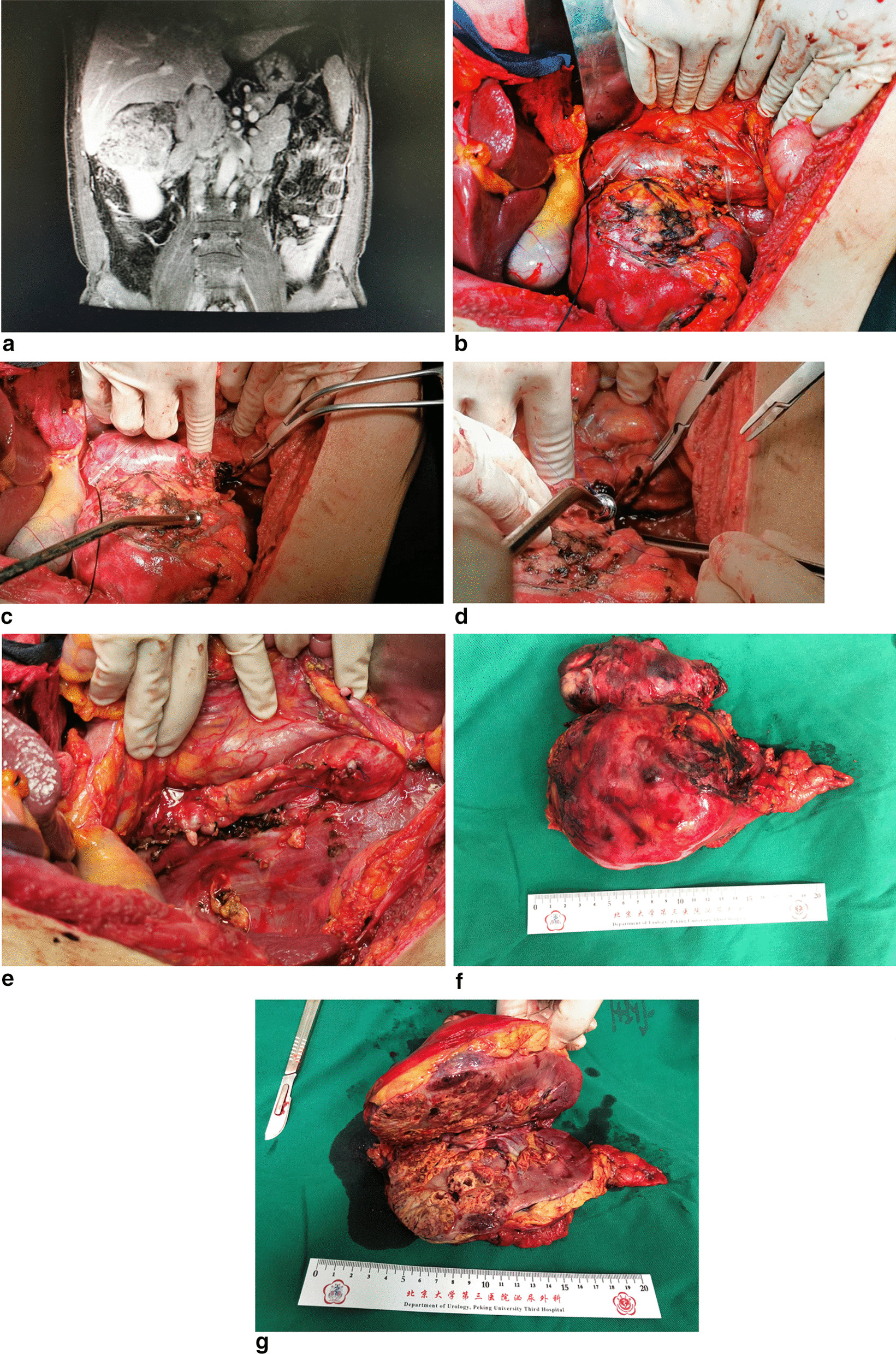
Typical preoperative and intraoperative images for right filled-type tumor thrombus. **A** Abdominal enhanced MRI showed right renal cell carcinoma(RCC) with filled-type tumor thrombus (RFTT). The tumor thrombus invaded the left and right wall of inferior vena cava.The extent of IVC involvement was about 12 cm. **B** In open surgery, the left renal vein, the distal end and the proximal end of inferior vena cava were fully exposed. **C** The distal end, the proximal end of the inferior vena cava and the left renal vein were transected separately. The distal end of the tumor thrombus was complicated with bland thrombus. **D** The distal stump of inferior vena cava was sutured with 4–0 Prolene suture. **E** Segmental resection of the inferior vena cava was used. The distal end, the proximal end of the inferior vena cava and the left renal vein were transected separately and the vascular stumps were sutured with 4–0 Prolene suture. **F** The surgical specimens showed the involved inferior vena cava has been segmental resected with the tumor thrombus inside. **G** The right kidney was incised to show a yellowish brown renal tumor inside

During the operation, the left renal vein, the distal end and the proximal end of inferior vena cava were fully exposed. The inferior vena cava under the renal vein (the distal end) was blocked firstly, the left renal vein was blocked secondly, and the proximal end of the inferior vena cava was blocked at last. After incision of the wall of inferior vena cava, the tumor thrombus filled and completely blocked the wall of inferior vena cava. Tumor thrombus invaded the left renal vein entrance. The distal end, the proximal end of the inferior vena cava and the left renal vein were transected separately. The vascular stump was sutured with 4–0 Prolene suture. (Typical preoperative and intraoperative images are shown in Fig. 1A-G).

#### Right non-filled-type tumor thrombus (RNFTT)

During the operation, the wall of inferior vena cava was open, and the tumor thrombus invaded the wall of inferior vena cava near the right side, while the tumor thrombus did not invade the entrance of left renal vein. The inferior vena cava was obliquely interrupted, and the left renal vein outflow was reserved. The distal end of IVC was transected and the vascular stump was sutured with 4–0 Prolene suture.

#### Left filled-type tumor thrombus (LFTT)

Segmental resection of the inferior vena cava was used. The distal end, the proximal end of the inferior vena cava and the right renal vein were transected separately. The vascular stump was sutured with 4–0 Prolene suture. In general, the collateral circulation of the right kidney was not well established. Therefore, this kind of operation was easy to lead to renal insufficiency.

#### Left non-filled-type tumor thrombus (LNFTT)

The tumor thrombus invaded the wall of inferior vena cava near the left side, while the tumor thrombus did not invade the entrance of right renal vein. The inferior vena cava was obliquely interrupted, and the right renal vein outflow was reserved. The distal end of inferior vena cava was transected and the vascular stump was sutured with 4–0 Prolene suture.

### Postoperative complications and follow-up

The Clavien-Dindo classification system [9] was used to evaluate the postoperative complications. Grade ≥ 3 was defined as major complications [10]. The patients were followed up at 3 months postoperatively, every 6 month during the first 5 years and every year after 5 years.

### Statistical analysis

For continuous variables, the Mann–Whitney U test was used. For categorical variables, Chi-square tests were used. Kaplan–Meier plots were performed to evaluate the influence of interruption of IVC on overall survival and cancer specific survival. Statistical analysis was performed using SPSS 24.0. A P value < 0.05 was considered statistically significant.

## Results

A total of 103 patients were identified. Patients’ clinical and pathological data were presented in Table 1. There were 77 males (74.8%) and 26 females (25.2%) with the median age of 60 years old. There were 75 cases (72.8%) with right tumors and 28 cases (27.2%) with left tumors. According to the Mayo classification, 67 cases (65.0%) were level II thrombus, 19 cases (18.5%) were level III thrombus, and 17 cases (16.5%) were level IV thrombus. Pulmonary embolism was found in 5 cases (4.9%) before operation.

**Table 1:** Clinicopathological characteristics of all patients

Characteristic	N = 103
Gender, n (%)	
Male	77 (74.8)
Female	26 (25.2)
Age at surgery, years	60 (53–65)
BMI, kg/m^2^	23.4 (21.1–26.0)
Side, n (%)	
Right	75 (72.8)
Left	28 (27.2)
Tumor diameter, cm	8.3(6.4–10.5)
ASA score, n (%)	
1	5 (4.9)
2	77 (74.7)
3	21 (20.4)
VTT level, n (%)	
II	67 (65.0)
III	19 (18.5)
IV	17 (16.5)
Preoperative PE, n (%)	5 (4.9)
Concomitant bland thrombus, n (%)	33 (32.0)
Preoperative lab values	
Creatinine, μmmol/L	92 (81–112)
Hemoglobin, g/L	119 (107–126)
Histological type, n (%)	
Clear cell	86 (83.5)
Non-clear cell	17 (16.5)
Nuclear grade	
I-II	33 (32.0)
III-IV	70 (68.0)
Pathologic T stage, n (%)	
T3b	70 (68.0)
T3c	28 (27.2)
T4	5 (4.8)
Lymph node enlargement present, n (%)	67 (65.0)
Distant metastasis present, n (%)	29 (28.2)

Continuous variables were presented as median (interquartile range)

Patients were divided into two groups with 32 cases (31.1%) underwent IVC interruption (Group 1) while 71 cases (68.9%) did not underwent IVC interruption (Group 2). The comparative information of the two groups was shown in Table 2. When comparing the perioperative data between the two groups, we found that patients in Group 1 had significantly longer median operative time (424 vs 362 min, P = 0.021), higher median blood loss (2450 vs 1000 ml, P = 0.001), higher median transfusion requirement (1600 vs 400 ml, P < 0.001), longer median postoperative hospital stays (13 vs 9 days, P = 0.004) and an overall postoperative complication rate (81.3% vs 47.9%, P = 0.001), but no statistically significant difference in Serum Creatinine at 7 days postoperatively (100 vs 101 μmmol/L, P = 0.8) and major complication rate (18.8% vs 12.7%, P = 0.7). However, comparison of baseline characteristics showed that there was significant difference in gender, ASA score, preoperative serum creatinine between the two groups. In order to eliminate the interference of these factors, a propensity score based matching method was used. We successfully matched 29 patients who underwent IVC interruption to 29 patients without this procedure in 1:1 ratio. No significant differences existed in baseline characteristics between the groups. The comparison of perioperative data showed that patients who underwent IVC interruption had significantly longer median postoperative hospital stays (13 vs 9 days, P = 0.022) and a higher overall postoperative complication rate (79.3% vs 51.7%, P = 0.027), but no statistically significant difference in median operative time (426 vs 387 min, P = 0.3), median blood loss (2400 vs 2000 ml, P = 0.2), median transfusion requirement (1600 vs 1200 ml, P = 0.087), median serum creatinine at 7 days postoperatively (99 vs 103 μmmol/L, P = 0.8) and major complication rate (17.2% vs 10.3%, P = 0.4).

**Table 2:** Comparison of baseline characteristics and perioperative outcomes between patients with IVC interruption or not

		Before Matching			After Matching		
Variables	All patients	Interruption of IVC	No interruption of IVC	P value	Interruption of IVC	No interruption of IVC	P value
No. of patients, n (%)	103 (100%)	32 (31.1)	71 (68.9)	–	29 (50.0)	29 (50.0)	–
Gender, n (%)				0.046			0.2
Male	77 (74.8)	28 (87.5)	49 (69.0)		25 (86.2)	21 (72.4)	
Female	26 (25.2)	4 (12.5)	22 (31.0)		4 (13.8)	8 (27.6)	
Age at surgery, years	60 (53–65)	61 (53–67)	60 (52–64)	0.7	61 (53–67)	62 (55–67)	0.7
BMI, kg/m^2^	23.4 (21.1–26.0)	24.1 (22.0–26.6)	23.0 (20.5–25.8)	0.078	23.9 (21.8–26.3)	22.9 (20.7–25.4)	0.3
Side, n (%)				0.9			0.4
Right	75 (72.8)	23 (71.9)	52 (73.2)		22 (75.9)	19 (65.5)	
Left	28 (27.2)	9 (28.1)	19 (26.8)		7 (24.1)	10 (34.5)	
Local symptoms	103 (73.8)	23 (71.9)	53 (74.6)	0.8	21 (72.4)	18 (62.1)	0.4
Systemic symptoms	34 (33.0)	11 (34.4)	23 (32.4)	0.8	10 (34.5)	13 (44.8)	0.4
Tumor diameter, cm	8.3(6.4–10.5)	7.7 (5.6–10.3)	8.4 (6.6–10.9)	0.3	7.7 (5.6–10.2)	7.0 (6.3–9.0)	0.8
ASA score, n (%)				0.027			0.6
1	5 (4.9)	0 (0.0)	5 (7.0)		0 (0.0)	0 (0.0)	
2	77 (74.7)	21 (65.6)	56 (78.9)		19 (65.5)	21 (72.4)	
3	21 (20.4)	11 (34.4)	10 (14.1)		10 (34.5)	8 (27.6)	
VTT level, n (%)				0.077			0.5
II	67 (65.0)	18 (56.3)	49 (69.0)		17 (58.6)	17 (58.6)	
III	19 (18.5)	10 (31.2)	9 (12.7)		8 (27.6)	5 (17.2)	
IV	17 (16.5)	4 (12.5)	13 (18.3)		4 (13.8)	7 (24.1)	
Preoperative lab values							
Creatinine, μmmol/L	92 (81–112)	110 (92–116)	89 (76–106)	0.002	110 (91–115)	91 (80–115)	0.072
Hemoglobin, g/L	119 (107–126)	117 (102–123)	120 (109–133)	0.15	118 (103–124)	111 (90–126)	0.7
Operative time, min	387 (312–476)	424 (375–508)	362 (288–471)	0.021	426 (375–507)	387 (300–502)	0.3
Estimated blood loss, ml	1500 (500–3000)	2450 (1350–4000)	1000 (300–2800)	0.001	2400 (1350–4000)	2000 (800–3000)	0.2
Blood transfusion, ml	1200 (0–2000)	1600 (1200–2700)	400 (0–1600)	< 0.001	1600 (1200–2600)	1200 (400–2000)	0.087
Serum Creatinine at 7 days postoperatively, μmmol/L	100 (81–121)	100 (86–119)	101 (79–123)	0.8	99 (87–118)	103 (81–117)	0.8
Postoperative hospital stay, days	10 (8–14)	13 (11–14)	9 (7–14)	0.004	13 (10–14)	9 (8–14)	0.022
Overall complication, n (%)	60 (58.3)	26 (81.3)	34 (47.9)	0.001	23 (79.3)	15 (51.7)	0.027
Major complication, n (%)	14 (13.7)	5 (18.8)	9 (12.7)	0.7	5 (17.2)	3 (10.3)	0.4

Perioperative complications of patients undergoing IVC interruption were described in Table 3. Common complications included postoperative transfusion in 10 cases, deep venous thrombosis in 8 cases, edema of bilateral lower limbs or scrotum occurred in 7 cases and infection in 6 cases. In addition, a few patients had the following perioperative complications, such as renal dysfunction/failure, respiratory complication, hepatic inadequacy, lymphorrhagia, ileas, cardiac complication (arrhythmia, heart failure), coagulation disorder. Only 1 patient experienced perioperative mortality.

**Table 3:** Perioperative complications of patients undergoing IVC interruption

Type of Complications	N
Edema of bilateral lower limbs or scrotum	7
Deep venous thrombosis	8
Infection	6
Renal dysfunction/failure	2/2
Postoperative transfusion	10
Respiratory complication (pleural effusion, pulmonary atelectasis, respiratory failure)	3
Hepatic insufficiency	2
Lymphorrhagia	2
Ileas	3
Cardiac complication (arrhythmia, heart failure)	3
Coagulation disorder	2
Perioperative mortality	1

**Fig2:**
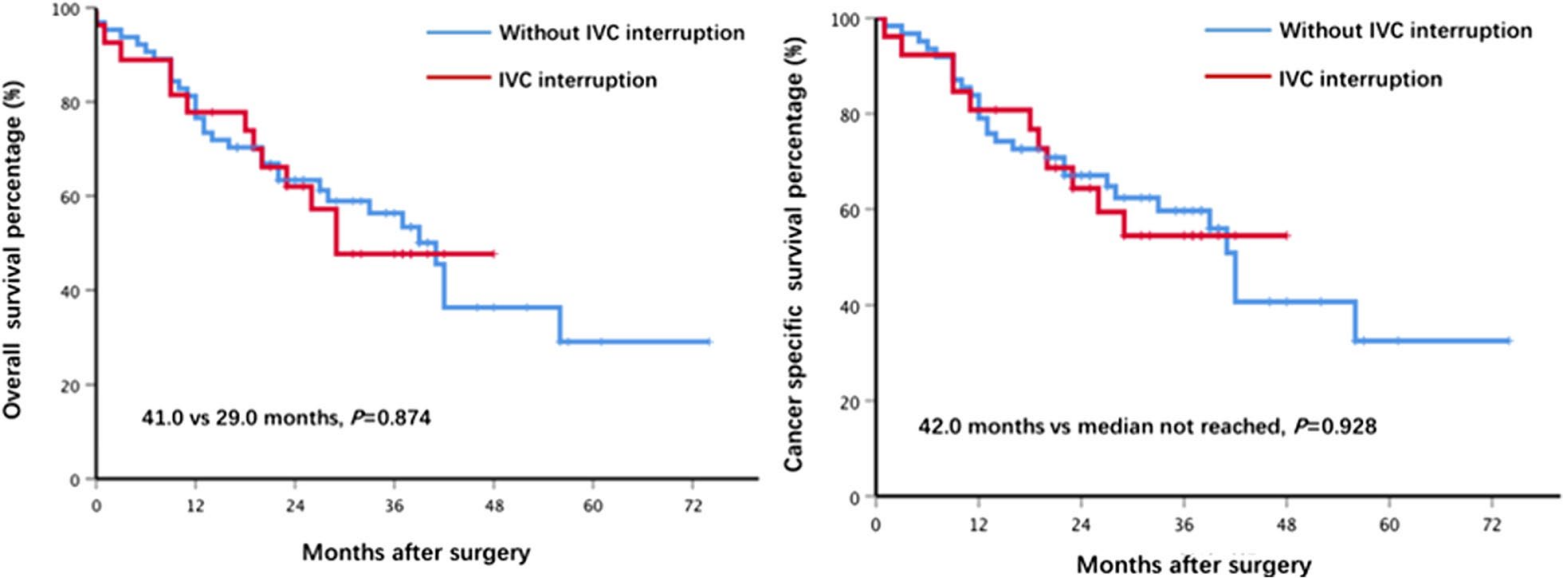
Overall survival and cancer specific survival between interruption of IVC group. and non-interruption of IVC group

We further compared the prognosis between the two groups in Fig. 2. We found that no significant difference existed in median overall survival (41 vs 29 months, P = 0.9) and median cancer specific survival (42 months vs median not reached, P = 0.9) between the two groups.

Different sides and shapes of tumor thrombus need different operative procedure of IVC interruption. According to the side and shape of tumor thrombus, it could be divided into four categories with different surgical procedures. There were 15 cases (46.9%) with right filled-type tumor thrombus (RFTT). In this category, the tumor thrombus filled and completely blocked the IVC. Tumor thrombus invaded the left renal vein entrance. The distal end, the proximal end of the inferior vena cava and the left renal vein were transected separately without left renal outflow tract. There were 8 cases (25.0%) with right non-filled-type tumor thrombus (RNFTT). In this category, the tumor thrombus invaded the wall of IVC near the right side, while the tumor thrombus did not invade the entrance of left renal vein. The inferior vena cava was obliquely cut, and the left renal vein outflow channel was reserved to let the left renal blood flow back into the IVC. There were 1 case (3.1%) with left filled-type tumor thrombus (LFTT). In this category, tumor thrombus invaded the right renal vein entrance. The distal end, the proximal end of the inferior vena cava and the right renal vein were transected separately without right renal outflow tract. There were 8 case (25.0%) with left non-filled-type tumor thrombus (LNFTT). In this category, the tumor thrombus invaded the wall of IVC near the left side, while the tumor thrombus did not invade the entrance of right renal vein. The IVC was obliquely cut, and the right renal vein outflow channel was reserved to let the left renal blood flow back into the IVC. Comparison of baseline characteristics and perioperative outcomes between patients with different IVC interruption methods was shown in Table 4.

**Table 4:** Comparison of baseline characteristics and perioperative outcomes between patients with different IVC interruption methods

Variables	Right RCC without left renal outflow tract	Right RCC with left renal outflow tract	Left RCC without right renal outflow tract	Left RCC with right renal outflow tract
Surgical indications	Right filled-type tumor thrombus (RFTT)	Right non-filled-type tumor thrombus (RNFTT)	Left filled-type tumor thrombus (LFTT)	Left non-filled-type tumor thrombus (LNFTT)
Surgical procedures (comparison of preoperative and postoperative)				
No. of patients	15 (46.9)	8 (25.0)	1 (3.1)	8 (25.0)
Gender, n (%)				
Male	13 (86.7)	8 (100)	1(100)	6 (75)
Female	2 (13.3)	0 (0)	0 (0)	2 (25)
Age at surgery, years	60 (53–67)	66.5 (62.5–72.5)	58	52.5 (47.3–59.5)
BMI, kg/m^2^	24.8 (22.5–26.6)	23.4 (21.6–28.5)	20.8	24.4(21.7–28.0)
Local symptoms, n (%)	12 (80)	4 (50)	1 (100)	4 (50)
Systemic symptoms, n (%)	3 (20)	4 (50)	0 (0)	4 (50)
Tumor diameter, cm	8.3 (6.8–10.3)	6.0 (3.8–9.3)	13.0	6.1 (5.6–14.5)
ASA score, n (%)				
2	9 (60)	5 (62.5)	1 (100)	6 (75)
3	6 (40)	3 (37.5)	0 (0)	2 (25)
VTT level, n (%)				
II	6 (40)	5 (67.5)	1 (100)	6 (75)
III	8 (53.3)	1 (12.5)	0 (0)	1 (12.5)
IV	1 (6.7)	2 (25)	0 (0)	1 (12.5)
Preoperative lab values				
Creatinine, μmmol/L	108 (85–113)	112 (104–119)	120.0	97(89–112)
Hemoglobin, g/L	116 (107–125)	106.5 (95.8–121.8)	123	119 (100–121)
Operative time, min	379 (372–426)	485.5 (326–554.8)	328	510 (403–538)
Estimated blood loss, ml	2000 (1500–2800)	2700 (500–5750)	2500	4050 (1725–4500)
Blood transfusion, ml	1600 (1200–2000)	1600 (400–3600)	1200	2400 (900–3550)
Serum Creatinine at 7 days postoperatively, μmmol/L	99 (87–116)	113 (93.8–132.5)	1002	87.5 (68–109)
Postoperative hospital stay, days	13 (12–14)	12 (9, 13)	13	13 (11–18)
Overall complication, n (%)	12 (80)	7 (87.5)	1 (100)	6 (75)
Major complication, n (%)	2 (13.3)	1 (12.5)	1 (100)	1 (12.5)

## Discussion

Radical nephrectomy and IVC tumor thrombectomy could effectively improve the prognosis of patients. For patients with IVC wall invasion, it was necessary to remove the involved vessel wall to achieve the goal of complete removal of all tumor burden. Segmental resection and interruption of the IVC was necessary when the tumor thrombus invaded the wall of the IVC extensively, and was anatomically feasible with a good recovery of renal function [11]. The collateral vessels around the IVC from the renal vein to the common iliac vein, could ensure the venous return of pelvic organs and lower limbs. The collateral circulation of IVC in the lower renal segment mainly depends on the inferior abdominal vein, lumbar vein collateral, vertebral vein plexus, azygos vein system and superficial abdominal wall vein [12]. In this study, the right or left filled type tumor thrombus (RFTT) was performed with the above methods. For other patients with small invasion area of IVC wall, the IVC was obliquely interrupted, and the left renal vein outflow was reserved to let the healthy renal blood flow back into the IVC. In this study, we used the above methods for right or left non-filled type tumor thrombus (NFTTT). This method could reduce the incidence of renal insufficiency, edema of lower limbs, varicocele and so on.

The choice of surgical method depended largely on the extent and location of tumor thrombus invading vascular wall. MRI scan could be used to determine the length of tumor thrombus and judge whether the tumor thrombus invades the IVC wall. The sensitivity and specificity of MRI in the diagnosis of tumor thrombus invasion into the inferior vena cava wall were 92.3% and 86.4%, respectively [13]. The imaging signs of invasion of inferior vena cava wall revealed by preoperative abdominal MRI scanning included: (1) rough and unsmooth wall of IVC; (2) large diameter of IVC vessel; (3) edema zone on the outside of IVC wall; (4) irregular shape of tumor thrombus [14]. Some scholars believed that the diameter of IVC greater than 40 mm in preoperative abdominal CT was a risk factor for invasion of IVC wall by tumor thrombus [15]. Contrast-enhanced ultrasound in patients with wall invasion showed that the tumor thrombus was enhanced synchronously with the IVC wall and the continuity of the IVC wall was lost, and its sensitivity and specificity were 93.1% and 93.5% [16]. Preoperative MRI in patients with wall invasion showed that the anteroposterior diameter of the IVC greater than 18 mm or the diameter of the renal vein entering the IVC greater than 14 mm, and the sensitivity of this method was 90% [17].

Although preoperative imaging examination could determine whether the tumor thrombus invaded the wall to a certain extent, intraoperative visual inspection was still an accurate and effective method. The signs of invasion during operation were as follows: rough and unsmooth vascular wall, white color after incision of IVC wall; hard texture of palpation and poor elasticity of blood vessels. Vena cavography could judge the obstruction of tumor thrombus and showed the establishment of venous collateral circulation. Since cavography is invasive and costly, it is rarely preferred [18]. This examination was not routinely carried out in our center. At present, there is no strict and accurate definition of tumor thrombus invading blood vessel wall. Our center integrates preoperative imaging findings, intraoperative inspections, and postoperative pathological results to comprehensively judge.

Interruption of IVC was quite different from that of left and right renal cell carcinoma [19]. We believe that tumor thrombus should be divided into four categories, and different sides and shapes of renal tumor thrombus need different operative procedure of IVC interruption. For right or left non-filled-type tumor thrombus, contralateral renal vein outflow tract could be reserved if there is no tumor thrombus invasion at the entrance of renal vein into IVC. Interruption of IVC is feasible for right filled-type tumor thrombus. The distal end, the proximal end of IVC and the left renal vein can be transected separately. But for left filled type tumor thrombus, segmental reservation should be avoided as far as possible to avoid renal congestion because sufficient collateral circulation could not be established due to the small and few branches of the right renal vein. Therefore, if there was no tumor thrombus invading the right renal vein, the IVC could be cut off obliquely and the outflow tract of right renal vein could be reserved. Only one patient with left filled-type tumor thrombus (LFTT) and segmental resection of the IVC was used.The distal end, the proximal end of IVC and the right renal vein were transected separately. The collateral circulation of the right kidney was not well established. Therefore, this kind of operation was easy to lead to renal insufficiency [20]. Postoperative edema of both lower limbs (Clavien grade II complication) occurred. With the establishment of collateral circulation, the above symptoms gradually disappeared.

The limitations of this study were as follows: This study was a retrospective study and was a single-center small sample size study. Multi-center prospective studies with a larger sample size are needed in the further.The follow-up time was short and long-term follow-up studies with more cases were needed, and the length of IVC resection was not accurately measured in the surgical records.

## Conclusions

IVC interruption as an intraoperative technique to handle extensive IVC wall invasion or unresectable infrarenal bland thrombus, will increase the incidence of overall postoperative complications, but not the risk of major postoperative complications. Tumor thrombus should be divided into four categories, and different sides and shapes of renal tumor thrombus need different operative procedure of IVC interruption. For right or left non-filled-type tumor thrombus, contralateral renal vein outflow tract could be reserved if there is no tumor thrombus invasion at the entrance of renal vein into IVC. Interruption of IVC is feasible for right filled-type tumor thrombus. The distal end, the proximal end of IVC and the left renal vein can be transected separately. But for left filled type tumor thrombus, segmental reservation should be avoided as far as possible to avoid renal congestion.

## Data Availability

The datasets used and/or analysed during the current study are available from the corresponding author on reasonable request.. The e-mail address: malulinpku@163.com (Lulin Ma).

## Abbreviations

IVC: Inferior vena cava
RFTT: Right filled-type tumor thrombus
RNFTT: Right non-filled-type tumor thrombus
LFTT: Left filled-type tumor thrombus
LNFTT: Left non-filled-type tumor thrombus
RCC: Renal cell carcinoma
